# Human-Machine Interface for the Control of Multi-Function Systems Based on Electrocutaneous Menu: Application to Multi-Grasp Prosthetic Hands

**DOI:** 10.1371/journal.pone.0127528

**Published:** 2015-06-12

**Authors:** Jose Gonzalez-Vargas, Strahinja Dosen, Sebastian Amsuess, Wenwei Yu, Dario Farina

**Affiliations:** 1 Center for Frontier Medical Engineering, Chiba University, Chiba, Japan; 2 Department of Neurorehabilitation, University Medical Göttingen, Göttingen, Germany; University of Chicago, UNITED STATES

## Abstract

Modern assistive devices are very sophisticated systems with multiple degrees of freedom. However, an effective and user-friendly control of these systems is still an open problem since conventional human-machine interfaces (HMI) cannot easily accommodate the system’s complexity. In HMIs, the user is responsible for generating unique patterns of command signals directly triggering the device functions. This approach can be difficult to implement when there are many functions (necessitating many command patterns) and/or the user has a considerable impairment (limited number of available signal sources). In this study, we propose a novel concept for a general-purpose HMI where the controller and the user communicate bidirectionally to select the desired function. The system first presents possible choices to the user via electro-tactile stimulation; the user then acknowledges the desired choice by generating a single command signal. Therefore, the proposed approach simplifies the user communication interface (one signal to generate), decoding (one signal to recognize), and allows selecting from a number of options. To demonstrate the new concept the method was used in one particular application, namely, to implement the control of all the relevant functions in a state of the art commercial prosthetic hand without using any myoelectric channels. We performed experiments in healthy subjects and with one amputee to test the feasibility of the novel approach. The results showed that the performance of the novel HMI concept was comparable or, for some outcome measures, better than the classic myoelectric interfaces. The presented approach has a general applicability and the obtained results point out that it could be used to operate various assistive systems (e.g., prosthesis vs. wheelchair), or it could be integrated into other control schemes (e.g., myoelectric control, brain-machine interfaces) in order to improve the usability of existing low-bandwidth HMIs.

## Introduction

An ideal human-machine interface (HMI) should allow a consistent, intuitive and simple control of a multi-function system with minimal user training. In addition, it should also integrate a feedback channel informing the user about the state of the device, thereby closing the control loop. Designing an HMI with these characteristics has been a challenging task, especially in rehabilitation engineering where an assistive system is used by patients with impaired motor and sensory capacities.

On the other hand, modern assistive devices are becoming increasingly sophisticated mechanical and electronic systems, designed for flexibility and equipped with multiple functions. For example, reaching and grasping, which are especially complex motor tasks characterized by dexterous movements around multiple degrees of freedom (DoF), can be assisted/restored using arm or hand exoskeletons [1,2] neuroprostheses [3], hybrid systems [3,4], and robotic prostheses [5]. Due to the maturation of the technology, these systems are nowadays designed to match as close as possible the capabilities of the human motor system. Modern hand prostheses, for example, can implement more than 10 grip types [6,7], while some arm prostheses [8] replicate closely the full kinematic chain of the human arm (e.g., 18 out of 22 DoFs). These designs demonstrate that various technological challenges (size, power, many DoFs) are being successfully overcome, but the user-friendly control of these complex devices still remains an open problem.

Traditionally, HMIs are designed as unidirectional signal processing chains. The user generates command signals, while the HMI operates as a decoder, capturing the signals and estimating the user movement intention [9]. In this framework, the user has to provide distinct patterns of activity to trigger different functions supported by the device. Therefore, to accommodate more functions, the user needs to produce more commands in a consistent manner. At the same time, the decoding performance decreases as the number of classes to be discriminated increases. Implementing this classic approach to HMI becomes particularly challenging in patients with a high level disability, since the availability of active signal sources can dramatically decrease [10,11]. Finally, some signals and decoding methods have intrinsically low bandwidth, and are therefore capable of generating only a limited number of discriminable commands even in healthy subjects (e.g., motor imagery brain computer interfacing) [12]. In the field of brain computer interfacing, the P300 evoked potential paradigm has been successfully used to increase the number of commands. In this approach subject focuses his/her attention to an infrequent sensory stimulus and this triggers an involuntary neural response, which is detected by the system and used to select a command [13]. Therefore, the user does not generate the command signals directly, but attends to the stimulus and waits for the system to detect the neural event. Different sensory modalities (visual, tactile and auditory) has been tested for this purpose, and the approach have been applied for spelling words [14], wheelchair control [15] as well as game playing [16].

In order to improve these HMI researchers have closed the loop by using various sensory feedback methods. Closing the loop allows a simple, but limited bilateral communication between the assistive device and the user, in which the system shows the user the results due to the generated command. This way the user is able to monitor whether the movement intention was decoded properly by the HMI o not [3,17–19].

Contrary to the classic approach, in which the HMI awaits for the user to generate a proper signal pattern and then feedback the result, here we present a novel concept in which the desired function is selected using bidirectional communication between the controller and the user. Specifically, the HMI presents the available actions to the user in a cyclical manner through an electrotactile menu interface (EMI). When the user “feels” that the desired function is available for triggering, he/she generates a signal to acknowledge the selection of the currently active option. In this study, the selection signal was the start of the movement, which was detected using an inertial measurement unit. The system then executes the selected function, while the same EMI can also be used to provide feedback about the system’s state to the user, thus closing the control loop. The novelty of this approach is that it greatly simplifies the communication interface on the user side, i.e., he/she needs to be able to provide only a single acknowledgement input. Therefore the tactile menu operates as a multi-level selection multiplier allowing the user to trigger an arbitrary number of functions using this single input. In these scenarios, the decoding of user intentions is trivial since only one signal has to be recognized by the HMI, and the specific option is selected by generating the acknowledgement at a proper time (when that option is being presented by the EMI).

In principle, the proposed concept is general and can be applied to the control of any multi-function device, from home appliances to assistive systems (e.g., wheelchair). Different tactile stimulation technologies can be used to present the options (e.g., vibration motors) and the acknowledgement signal can come from different sources (e.g., electromyography or electroencephalography). The aim of the current study was to present the approach and demonstrate its feasibility by developing and testing one possible implementation (e.g., EMI + inertial units).

The control of grasping in a state-of-the-art active hand prosthesis was chosen as the context for the testing of the proposed HMI. This context was selected since modern myoelectric hand prostheses are capable of implementing several grasp types and therefore represent a relevant example of a multi-function device. Also, they are controlled using a standard, commercially and clinically accepted myoelectric HMI, which could thus be used as the relevant benchmark to compare the novel approach. Finally, using a prosthesis represents a rich context, which entails not only function selection, but also dynamic interaction through multiple phases, i.e., reaching for an object, grasping, lifting, manipulating and releasing. This multifaceted scenario was therefore convenient for exploring the full potential of the novel HMI, allowing its application in different forms (single and two-level menu), modes (menu and feedback), and multiple times during a single execution cycle. More specifically, the EMI was used to present a set of available grasp types and aperture sizes, while the acknowledgement signal was the initiation of the reaching movement detected by an inertial sensor. Furthermore, the automatic control of opening, closing, grasping force with feedback and releasing were also implemented using the novel HMI and its components without relying on any myoelectric control.

Importantly, it was not the aim of this study to show that the developed prototype is better than other solutions presented in literature for the control of multi degree of freedom prostheses. This field is characterized with a very active research which is motivated by a strong development of prosthetic technology, leading to sophisticated systems that in turn require the control interfaces capable of accommodating the emerging functionality. Some of the important recent developments such as robust pattern recognition [20–23], methods based on the invasive interventions [11], [24–26], and biologically inspired approaches [27–29]) are still in the research phase but can lead to advanced practical solutions in the future.

The proposed HMI in the current study should be regarded as one particular implementation of a method with a rather general applicability. In the context of prosthetics, it demonstrates a simple, non-invasive approach to the control of multi-DoF prostheses that is radically different from the usual solutions targeting this problem (i.e., myoelectric interfaces). As demonstrated in the study, it performs similarly and in some aspects better than the commercial state of the art proportional and sequential myoelectric control. Furthermore, it employs automatic operation in order to decrease the burden from the user. The prosthesis is operated by triggering a set of predefined “motor programs” by simply reaching for an object (shared control [17,30]). Therefore, the user can focus on the functional goal (i.e., grasping and manipulating) rather than the activation and performance of the grasp itself.

The novel method is envisioned not to replace but to improve the other available HMI systems. Specifically, it demonstrates an original and practical approach for the selection of multiple commands that could be easily integrated into the other control frameworks. The first prototype and favourable comparison with the existing commercial and clinical benchmark is a strong indication that such an approach could be indeed useful.

## Materials and Methods

### System components

The prototype system comprised the following components:

1. An 8-channel current-controlled stimulator (RehaStim, Hasomed Gmbh, DE) connected to a set of self-adhesive, disposable concentric electrodes (CoDe 501500, 4cm diameter, SpesMedica, IT). In the current study, only 4 channels have been used to implement the electrotactile menu system and feedback interface. Concentric electrodes comprised an inner active field (cathode) and an outer ring (anode). Low level electrical current pulses were delivered to the skin activating cutaneous afferents and eliciting well localized tactile sensations. The stimulation parameters (amplitude, pulse width and frequency) could be adjusted online from a host PC by sending commands via a USB connection.
2. Michelangelo hand prosthesis (Otto Bock HealthCare Products GmbH, AT) capable of implementing two grasp types (palmar and lateral) using proportional control of closing velocity and grasping force, and equipped with position and force sensors. The prosthesis was connected to the host PC using a Bluetooth interface. The host PC controlled the hand by sending commands and received the online sensor data at the sampling rate of 100 Hz.
3. An Inertial Measurement Unit (ZMP IMU-Z, ZMP INC., JP) comprising a 3-axis gyroscope (±500°/s at 12 bits resolution), accelerometer (±2g at 12 bits resolution), and magnetometer (±0.7 gauss at 12 bits resolution). The inertial data were sampled at 100 Hz and delivered to the host PC via a Bluetooth interface.
4. A standard Windows 7 desktop computer (dual core 2.4 GHz, 4 Gb RAM): was used to implementing the control software for the closed loop system running in Matlab 2012 (MathWorks, Natick, MA, USA).

**Fig 1:**
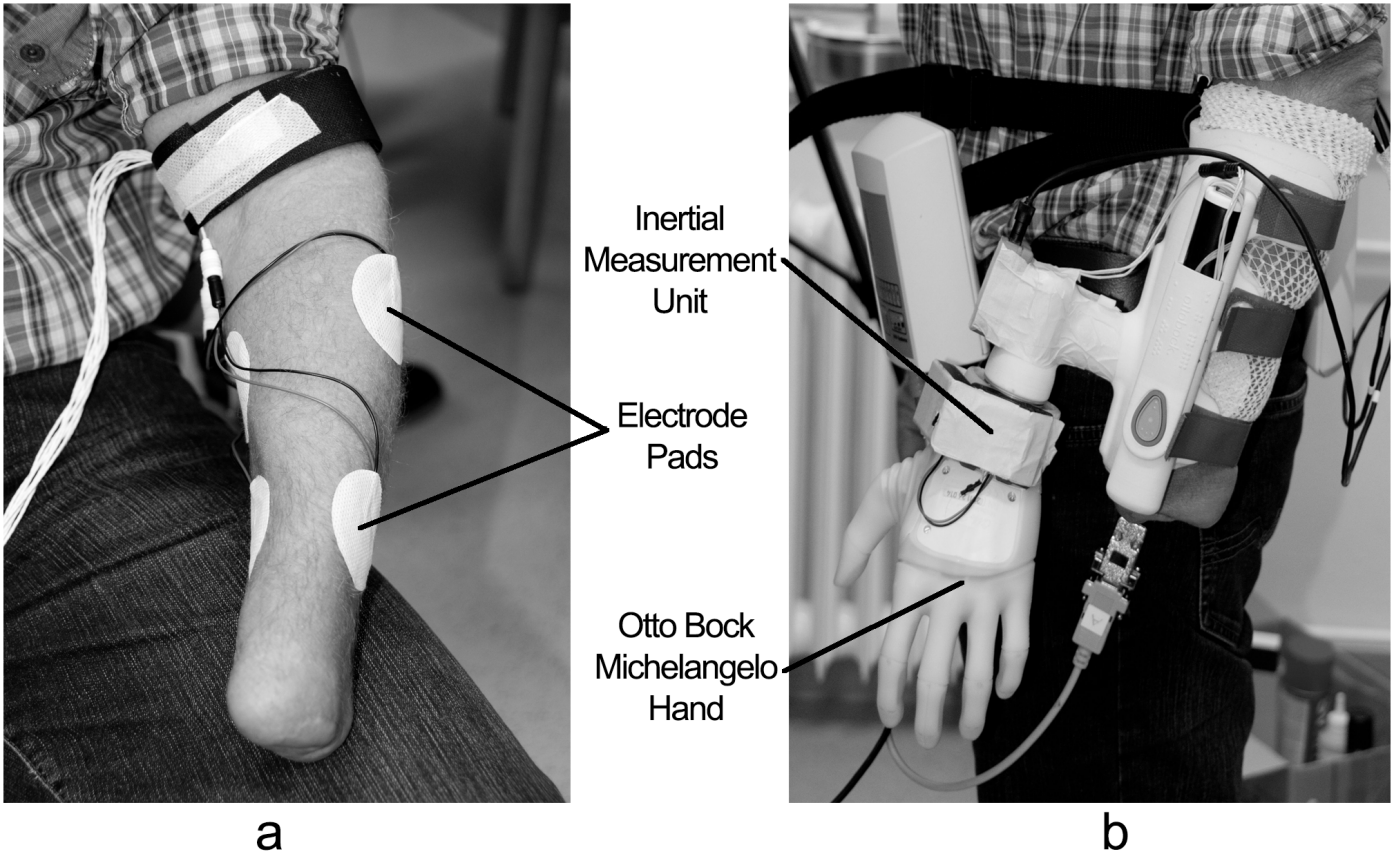
Hardware used to test the prototype. a) 4 Electrode Pads were placed on the forearm. b) Otto Bock Michelangelo hand was controlled using an Inertial Measurement Unit to detect subject’s motion and an electrocutaneous stimulator to implement the Menu Interface and the Feedback Interface.

The system was mounted on the subjects as shown in Fig 1. Four concentric electrodes were placed on the left forearm, two on the dorsal side and two on the volar side. At each side, one electrode was placed proximally and one distally from the elbow. The stimulation unit was attached to a waist belt. The prosthetic hand was mounted onto an orthopedic splint wearable for healthy subjects and amputees alike. The splint was strapped to the forearm using Velcro straps and the IMU was positioned onto the splint just below the hand attachment.

### Electrotactile Stimulation Settings

**Fig 2:**
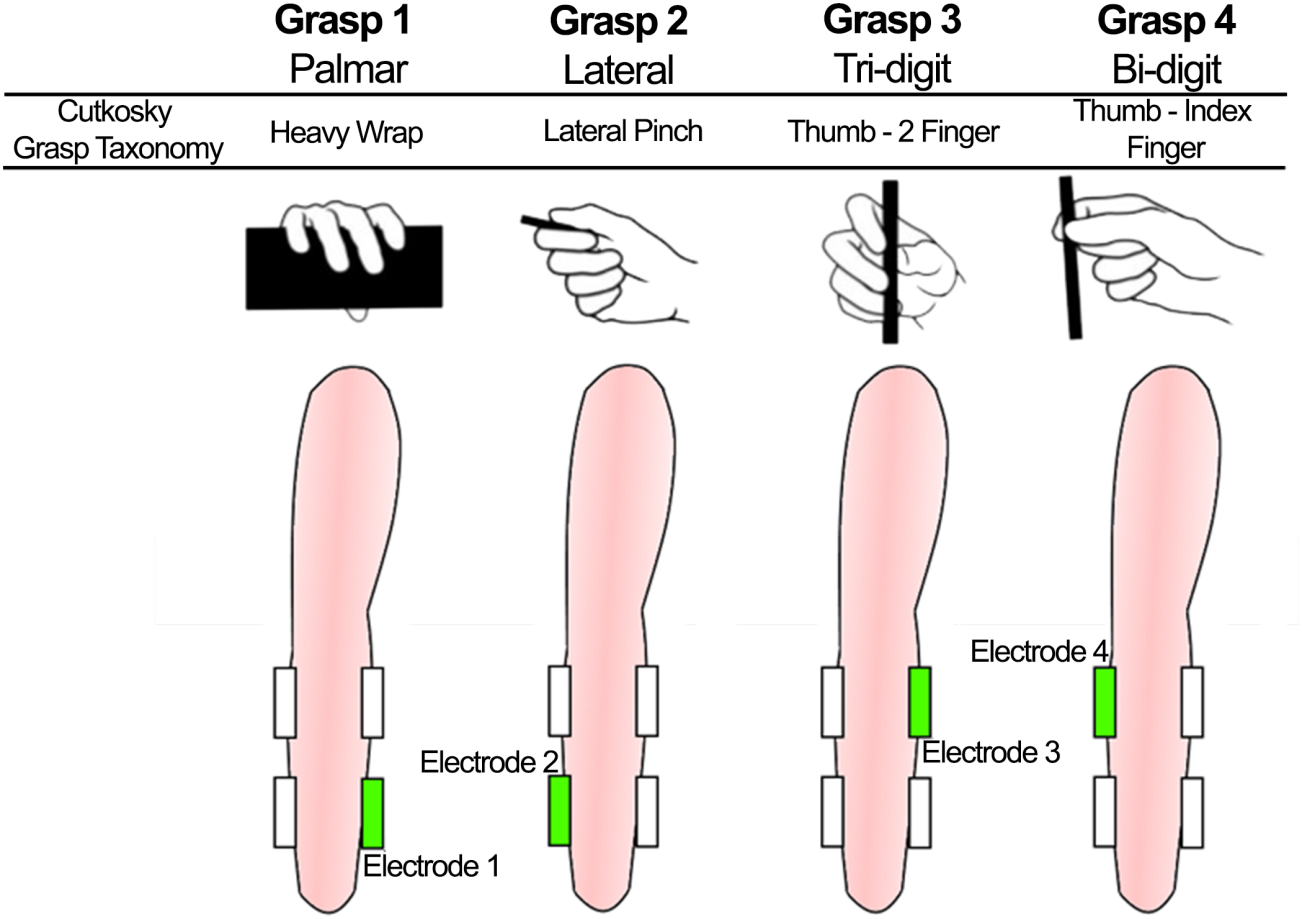
Electrode assignment to the grasps. Grasp 1: palmar was assigned to electrode 1 placed in the volar and distal side of the arm. Grasp 2: lateral was assigned to electrode 2 placed in the dorsal and distal side of the arm. Grasp 3: tri-digit was assigned to electrode 3 placed in the volar and proximal side of the arm. Grasp 4. Bi-digit was assigned to electrode 4 placed in the dorsal and proximal side of the arm. These grasps correspond to the heavy wrap, lateral pinch, thumb—2 fingers, and thumb—index finger from the grasp taxonomy proposed by Cutkosky et al in [36].

The electrotactile menu allowed the user to select the desired grasp type and size. The available grasp type and size combinations (menu options) were presented to the user by delivering electrotactile stimulation using a combination of spatial and intensity coding to represent the possible choices [31–34]. The activation of each electrode corresponded to a certain grasp type while the intensity of stimulation coded the size of the grasp, i.e., low intensity for a small grasp and high intensity for a large grasp. The low and high intensities were adjusted by setting the current amplitude to 1.2 × ST and 0.8 × PT, where ST and PT denote the sensation and pain thresholds, respectively. The thresholds were determined for each subject and electrode individually using the method of limits [35]. The pulse width and stimulation frequency were constant and set to 200 us and 50 Hz, respectively. This setup elicited the sensations that could be clearly felt and reliably discriminated (high vs. low) by the subjects. The electrodes were assigned to the grasps as in Fig 2. Grasp 1: palmar (electrode 1, volar and distal), Grasp 2: lateral (electrode 2, dorsal and distal), Grasp 3: tri-digit (electrode 3, volar and proximal), and Grasp 4: bi-digit pinch grasp (electrode 4, dorsal and proximal). These grasps correspond to the heavy wrap, lateral pinch, thumb—2 fingers, and thumb—index finger from the grasp taxonomy proposed by Cutkosky in [36]. Each menu option was active for 1 second. This particular arrangement and timing was implemented based on pilot tests to maximize discriminability, taking into account the limitations of electrotactile interfaces [31–34]. The electrodes were well separated (> 1 cm), placed on the opposite sides of the forearm (volar and dorsal), activated in the cross-pattern (dorsal-volar-dorsal-volar), and the stimulation was brief (1 s).

Overall, the subjects could select from a maximum of 8 options (4 grasp types × 2 grasp sizes). After selecting an option, the prosthetic hand would automatically preshape according to the selection. The use of the menu is described in detail in section II.C *System operation*. Two fixed grasp sizes were implemented in order to adapt the hand to different sizes of target objects and also to test the system performance with a larger number of options. The two grasp sizes were determined heuristically for each grasp type by considering the characteristics of the objects that are typically grasped using a specific grasp type (e.g. bi-digit pinch is used to grasp small objects). Due to its mechanical limitations, the Michelangelo prosthetic hand could not perform dedicated tri- and bi-digit grasps. Therefore the functionally equivalent palmar grasp mode was used with different apertures to simulate both of these pinch grasps.

### System Operation

**Fig 3:**
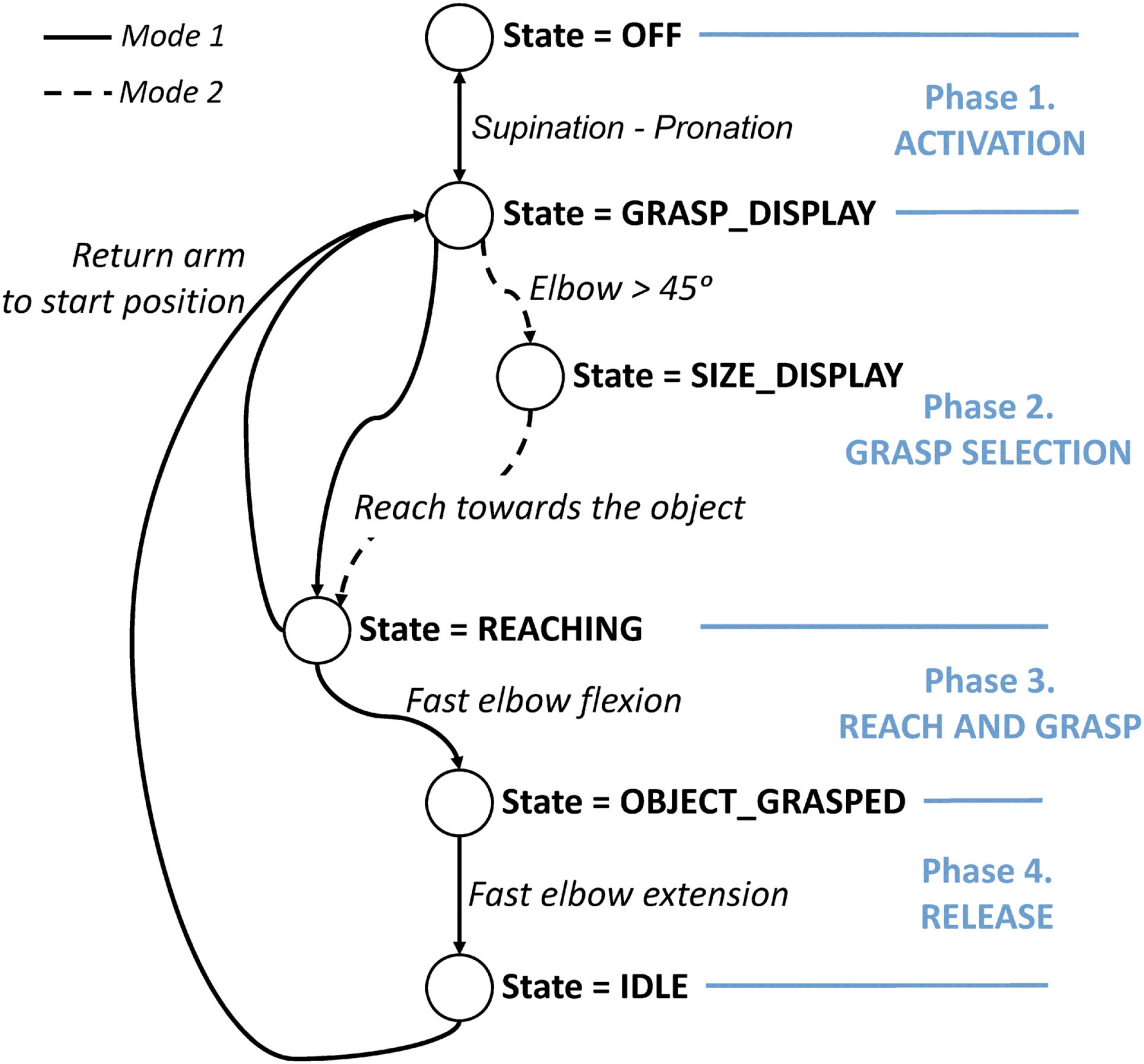
State machine controlling the operation of the prosthetic hand.

The operation of the prosthetic hand was divided into 4 phases, which were used to develop a state machine that allowed a complete control of the artificial limb (Fig 3). Each phase was characterized by well-defined actions that the subject had to perform in order to trigger the transition between the hand states. The subject’s movements were detected using the inertial sensors and the state transitions were governed by a set of IF-THEN rules, using the data from the x-axis of the accelerometer and the x, y and z axis of the gyroscope. These rules were handcrafted after analysing the data recorded during pilot experiments. Once constructed, the rules showed to be very robust since the same thresholds could be used for all subjects, i.e., it was not necessary to recalibrate the system for each individual. A mathematical description of the rules can be found in Appendix 1.

**Fig 4:**
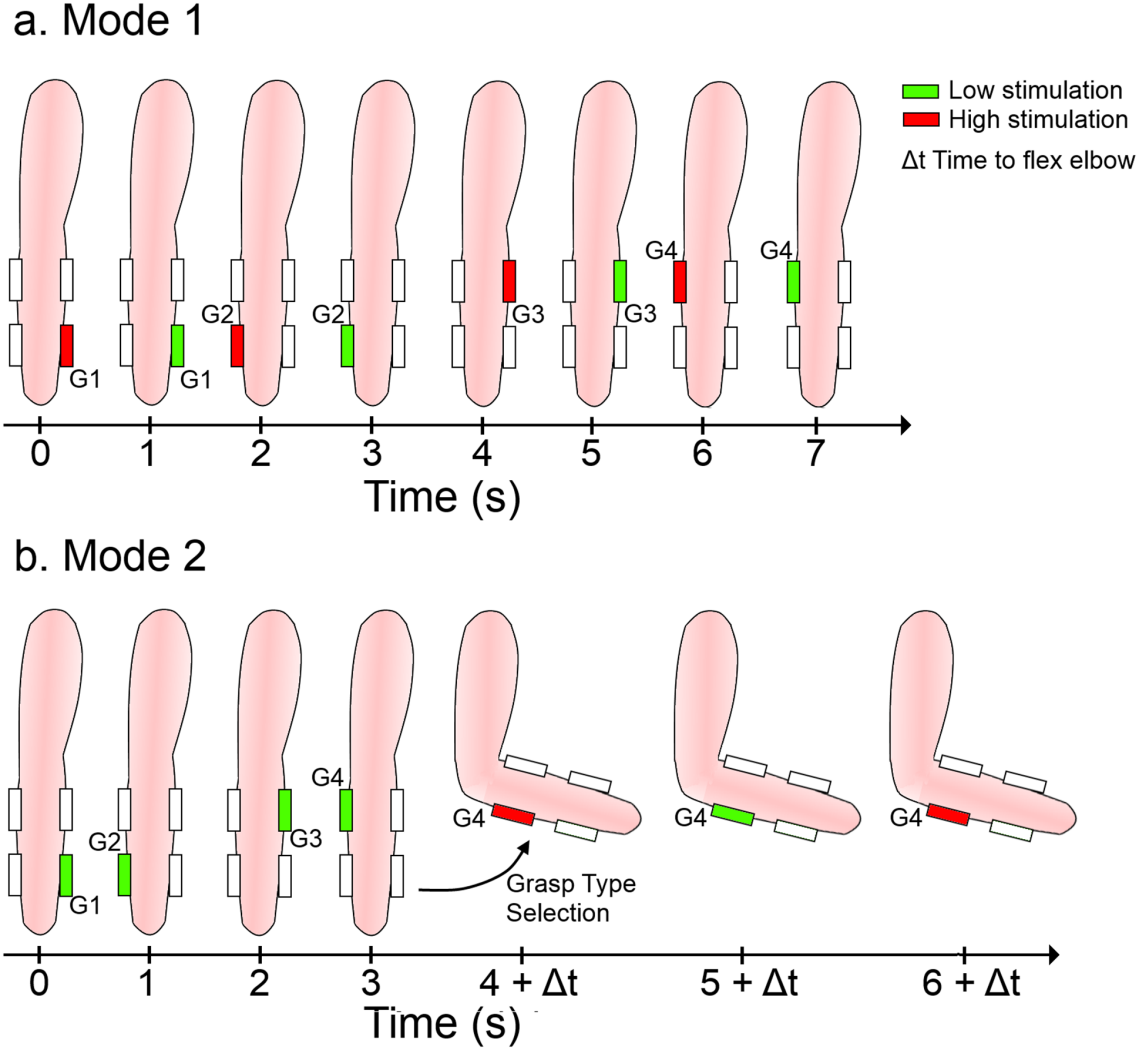
Menu interface modes. **a.** Mode 1, the grasp types and sizes were simultaneously presented to the user by sequentially activating the electrodes (grasp type), first at high (large size) and then at low intensity (small size). **b.** In Mode 2, only the grasp types were presented first, by activating the electrodes sequentially (low intensity), and when the grasp type was selected (elbow flexion), grasp sizes were presented by activating a single electrode cyclically at high and low intensity.

Each of the phases with the corresponding states and transitions are described in detail as follows:

1. System activation phase (ACTIVATION). The system was turned off by default (state = OFF), and therefore, the first step was the system activation (state = GRASP_DISPLAY). For this, the user had to perform a brief and small-amplitude supination and pronation motion of the forearm while the arm was placed in the starting position (time 0 in Fig 4). Repeating the same motion deactivated the control system (state = OFF). The assumption was that the user would turn on the system and start the electrotactile menu only when he/she actually intends to use the prosthesis.
2. Grasp type and size selection phase (GRASP SELECTION). Immediately after the system was turned on, the available grasp options were presented to the user via the EMI. We implemented two different control schemes (Fig 4) to explore possible differences in performance:
  - Mode 1 (single-step selection): the electrodes were sequentially activated to cycle through all the available grasp types and sizes. Specifically, electrode 1 at high intensity (palmar large) was activated first, then electrode 1 at low intensity (palmar small), then electrode 2 at high intensity (lateral large), etc. When the subject felt that the option representing the desired grasp type and size was active, he/she would simply start reaching for the target object. This stopped the electrotactile stimulation menu and triggered the transition into the REACHING state. The user was also able to cancel the selection by moving back to the starting position (returning to the GRASP_DISPLAY state). Mode 1 was a tactile equivalent of a single-level visual menu (i.e., all possible choices in the same list).
  - Mode 2 (two-step selection): the electrodes were activated sequentially at the low intensity cycling through the available grasp types, i.e., electrode 1 at low intensity was activated first (palmar grasp), then electrode 2 at low intensity (lateral grasp), electrode 3 at low intensity (tridigit grasp) etc. When the subject felt that the desired grasp type option was active, he/she should flex the elbow for more than 45° degrees, and this caused a transition into the SIZE_DISPLAY state. At this point, the electrocutaneous stimulation was continuously delivered through the electrode corresponding to the selected grasp type while the intensity was constantly changed between the high and the low to denote the size of the grasp (large and small, respectively). To select the desired size, the user would simply start reaching when the right intensity was felt, which triggered the transition into the REACHING state. Similarly to Mode 1, if the hand was moved to the starting position, the system was set back to the GRASP_DISPLAY state. Mode 2 was a tactile equivalent of a two-level visual menu (i.e., menu for the grasp types with the submenu for the sizes).
3. Reaching and grasping phase (REACH AND GRASP). While reaching for the desired object, the hand automatically preshaped to the selected grasp and started closing. When a small grasp was selected, the prosthetic hand would close at maximum speed until it reached a predefined target aperture suitable for grasping smaller objects. At this moment the hand would stop for 1 second and then continue closing at a slower speed. When a large grasp was selected the hand would start closing slowly, right from the beginning of the motion, so that the user had enough time to enclose a large object. These two schemes for the small and large grips allowed the users to achieve more natural and continuous reaching motion.
  When a contact with the object was made, the hand started increasing the force exerted on the object at a predefined constant rate (pilot tests). At the same time, the EMI changed to the feedback interface and the grasping force was conveyed to the subject by modulating the stimulation frequency from 1 Hz (minimum force) to 255Hz (maximum force). This allowed the subject to monitor the grip force that was being exerted on the object. When the subject judged that the appropriate grasping force was reached, only a short elbow flexion was needed to lock the grasp and consequently lift the object, prompting the system to move to the OBJECT_GRASPED state.
4. Release phase (RELEASE). Once the grip was locked, the user was able to move and manipulate the object. At this point, the feedback interface changed back to the EMI. However, this time the EMI indicated the possibility of releasing the grip. This was implemented to allow the user to hold and manipulate the object while avoiding an accidental release. Two channels (volar and dorsal distal electrode) were activated according to a specific time pattern: electrode 1 at low intensity (0.3s), short pause (0.3s), electrode 2 at low intensity (0.3s), long pause (1s), and repeat. In order to release the object, the subject had to synchronize a quick elbow extension so that it was performed during the activation of electrode 2. The activation of electrode 1 was cuing the subject to start the movement. Note that the movement (elbow extension) corresponds to a movement that is normally performed right before placing and releasing an object. This would put the state machine into the IDLE state, from which the subject could return to the state GRASP_DISPLAY by moving the arm back to the starting position.

## Experimental Settings

### Experimental protocol: Menu Interface

Twelve able-bodied subjects (8 male and 4 female, 25–35 years) and one subject (male 56 years, myoelectric prosthesis user) with a left wrist disarticulation participated in this study. The experiment was explained to the subjects, who signed an informed consent approved by the Ethics Committee of the University Medical Center Goettingen (no. 22/2/12). The sensation and pain thresholds for electrical stimulation were determined using the method of limits [32,35]. Next, the prosthetic hand was mounted to the left forearm using a custom made splint and the subjects were trained for about 15–20 minutes to familiarize with the system.

During the experiment, the subjects had to reach for and grasp a set of daily life objects, as described in Table 1. The general principle for assigning a grasp type and size to an object was explained to them (e.g., palmar large grasp was used for wide cylindrical objects etc.).

**Table 1:** Description and dimensions of objects used for the experiments with the assigned type and size of grasp.

Grasp Type	Objects	Large Size (mm)	Small Size (mm)
Palmar	Cylinders	110 > diameter > 60	diameter < 60
Lateral	Box, CD Box, a Key	70 > width > 30	width < 30
Tri-Digit	Long cubes	40 > width > 20	width < 20
Bi-Digit	Bottle Cap, small screw, small sphere.	40 > diameter > 20	diameter < 20

The objects were placed at six different locations: {Bottom, Top, Middle} × {Left, Right}. A cabinet with adjustable shelves was used to adjust the positions for each subject so that during grasping the forearm was roughly 0° (middle), inclined upward by 30° (top), and downward by 30° (bottom). Similarly, for the left and right positions, the arm had to be abducted or adducted by approximately 15°, respectively. These positions were selected to test the system’s robustness by emulating the way grasping is performed in daily life (i.e., grasping in different directions and heights). At the beginning of each trial, the subject was asked to stand still in front of the cabinet with the arms resting vertically (starting position). A random object was randomly placed in one of the six positions and the subject was asked the type and size of grasp he/she would select for this particular object. Then he/she was instructed by the experimenter to start performing the following movement sequence: turn the system on, select a grasp type and size, reach and grasp, lift the object and hold it for 2 to 3 seconds, and finally release the object back to its original position.

Four conditions were tested randomly:

- Mode 1, two grasps (M1G2). The single-step selection was used to choose between two grasp types (palmar and lateral) with two grasp sizes (4 options). This mode can be described by the following equation:
  $$t_{option}=T_{d}*N$$ (1)
  where *t*_*option*_ is the time elapsed before the option *N* in the menu becomes active, *T*_*d*_ is the time between options (fixed to 1s), and *N* = 1, 2, 3, 4 is the option number. This condition was implemented to assess the performance when the control system supported the functionality that was actually available in the Michelangelo hand.
- Mode 1, four grasps (M1G4). The single-step selection was used to choose between four grasp types with two grasp sizes (8 options). This mode can be also described by eq 1 with *N* = 1, 2 … 8. This condition was implemented to evaluate the control with more options available, as if the system was applied to control a dexterous hand with four grasp types. M1G4 was compared to M1G2 in order to assess the subject performance in Mode 1 when the menu was more complex (more options).
- Mode 2, four grasps (M2G4). The two-step selection was used to choose between the same number of grasp types and sizes as in M1G4. Our hypothesis was that this mode would allow a faster selection of the options in the menu, especially for the latter ones. This mode can be described by the following equation:
  $$t_{option}=T_{D}*\|\frac{N-1}{2}+\Delta t+even(N)$$ (2)
  where *T*_*D*_ is the time between the options (fixed to 1s), with *N* = 1, 2 … 8, Δt is the elbow flexion duration, and even(N) is a function returning 1 if *N* is an even number and 0 otherwise. If we assume that Δt is around 1 s, the waiting times (*t*_*option*_) in Mode 2 would be the same as in Mode 1 for options 1 and 2 and shorter for option 3 and higher, with the difference of 3 seconds for the last option in the menu. This condition was introduced to compare the two selection modes (M2G4 vs. M1G4).
- Mode 2, two grasps (M2G2). The two-step selection was used to choose between two grasp types (palmar and lateral) with two grasp sizes each (4 options). Due to time constrains, this condition was used only with the amputee (M1G2 vs. M2G2).

### Experimental protocol: Myoelectric Interface

The myoelectric control experiment was performed with 6 able-bodied subjects participating also in the experiment with the EMI. For this session, a commercially available prosthesis control kit from Otto Bock Healthcare Products GmbH (Vienna, Austria) was used. Two 13E200-50 double differential active electrodes were placed approximately 6–7 cm distally from the elbow on the forearm of the subject, one on the flexor and the other on the extensor muscles. The electrodes allowed proportional control of the closing and opening and grasping force of the prosthetic hand. The electrodes were connected to an AxonMaster 13E500 controller, which was used to process the EMG signals. The prosthesis was mounted in the same way as in the previous test. AxonMaster allowed selecting between two different grasp types (palmar and lateral) by performing a brief cocontraction (CoCo) of the flexor and extensor muscles. Each time a CoCo was performed, a short acoustic feedback was provided to the user (factory default setting).

In order to allow the selection of more options, we have emulated the Otto Bock controller on a personal computer and extended it to accommodate 4 grasps. The Otto Bock interface was still used for EMG acquisition.

Each subject was tested in the following modes:

- Four grasp types, audio feedback and grasp type resetting (G4AR). This condition was compared to M1G4 and M2G4. The time needed to select an option (activate a grasp *N*)in this mode is described by the following equation:

$$t_{option}=T_{cc}*N$$ (3)

where *T*_*cc*_ is the time taken by the subject to achieve a successful CoCo, with *N* = 1, 2, 3, 4. The latter means that the subject performed a CoCo and that this was successfully detected by the system.

- Four grasp types, audio feedback and no grasp type resetting (G4A). In this case the subject had to keep track of the currently active grasp. This condition was compared to M1G4 and M2G4. The time needed to select an option (activate a grasp *N*) in this mode is described by the following equation:

$$t_{option}=T_{cc}*(N-N_{last})$$ (4)

where *T*_*cc*_ is the time taken by the subject to achieve a successful CoCo, with *N* = 1, 2, 3, 4, and *N*_*last*_ = 1, 2, 3, 4 denotes the grasp achieved in the last trial (no resetting).

- Four grasp types, no audio feedback and no resetting (G4). The same as in the previous condition but with no acoustic feedback. Therefore, the subjects did not have the information if the CoCo was actually detected by the system. This condition was compared to M1G4 and M2G4 and is described also by eq 4.

At the beginning of the experiment, the subjects were trained for 15–20 minutes to operate the prosthesis using myoelectric control. The experimental task was the same as for the assessment of the EMI.

## Data Analysis

The following outcome measures were used:

1. Time needed To Activate a grasp (TTA): This measure evaluated the time needed for the successful selection and activation of a grasp. With the EMI, the subject had to wait for the correct option to be presented in order to activate the grasp (start reaching). Thus, the TTA was a function of the location of the grasp to be chosen in the cyclic menu. In the myoelectric control, the subjects had to generate correct number of successful CoCos. The TTA was used for comparing the speed in selecting functions using Menu and Myoelectric interface.
2. Grasp Selection Performance (GSP): Before starting the trial, the subjects were asked to state which grasp they intended to realize for the presented object. If the grasp that was actually selected in the trial was the same as the intended grasp, the grasp selection was deemed successful. This index evaluated the ability of subjects to correctly select the desired grasp.
3. Grasp Attempts (GA): In the EMI, the subject needed to realize a fast elbow flexion for the prosthetic hand to lock the grasp. In the myoelectric control the subject had to activate the flexor muscles to command the hand to close and grasp the object, and then relax the muscles to lock the grasp. There were cases when more than one attempt was needed for both interfaces in order to successfully activate the locking. Therefore, this index measured the average number of attempts per trial necessary to lock the grasp.
4. Grasp Performance (GP): This measure indicated how well in percent success rate the subjects were able to grasp an object. If the subject grasped and successfully lifted the target object from the support, the trial was deemed successful.
5. Release Attempts (RA): In the EMI, the subject needed to synchronize the elbow extension with the stimulation pattern, pacing the release action. In myoelectric control, the subjects had to activate the extensor muscles to start opening the prosthetic hand. There were cases when more than one attempt was needed to activate the hand and release the object. Therefore, this index measured the average number of attempts necessary to release an object.

In order to compare the data between the Myoelectric control and EMI, a Related-Samples Wilcoxon Signed Rank Test was used since the data were not normally distributed (Leven’s test). Moreover, a comparison of GA and RA between the different positions and the TTA for the different grasping type options was done using Friedman’s ANOVA test.

## Results

### Comparison between Mode 1 and Mode 2 of the Menu Interface

Table 2 shows the mean value and the standard deviation of the results obtained for all subjects. The GSP significantly decreased in M2G4 (p<0.05, r = 0.49 where r is the effect size). The subjects were therefore less successful when using Mode 2 to select the desired grasp. The GA and RA were similar in all conditions, since after the grasp selection, the other steps (grasp locking, object release) were performed in the same way. For M1G2, the data from 3 subjects could not be used (acquisition error).

**Table 2:** Mean values (and standard deviation) of the results obtained during Mode 1 for 2 grasps (M1G2), Mode 1 for 4 grasps (M1G4) and Mode 2 for 4 grasps (M2G4).

	M1G2	M1G4	M2G4
Grasp Selection Performance (%)	90.3(12.5)	92.1 (8.9)	85.6 (9.5)*
Grasp Attempts	1.1 (0.1)	1.1(0.1)	1.1(0.1)
Grasp Performance (%)	94.7(14.0)	87.3 (6.4)	86.1(8.5)
Release Attempts	1.2(0.1)	1.1 (0.4)	1.2 (0.1)

* p < 0.05 with respect to M1G4; Number of subjects = 12, grasped objects per subject = 16.

Table 3 shows the results obtained with the amputee, who only used the system in M1G2 and M2G2 due to time constrains. He easily understood the concept of the novel control method and learned to use the system very fast. The results demonstrated that he also achieved a very good performance. Again in Mode 1, the overall GSP was better than in Mode 2.

**Table 3:** Results obtained with the amputee subject during Mode 1 and Mode 2 for 2 grasps.

	M1G2	M2G2
Grasp Selection Performance (%)	100	88
Grasp Attempts	1.0	1.0
Grasp Performance (%)	100	100
Release Attempts	1.07	1.0

**Fig 5:**
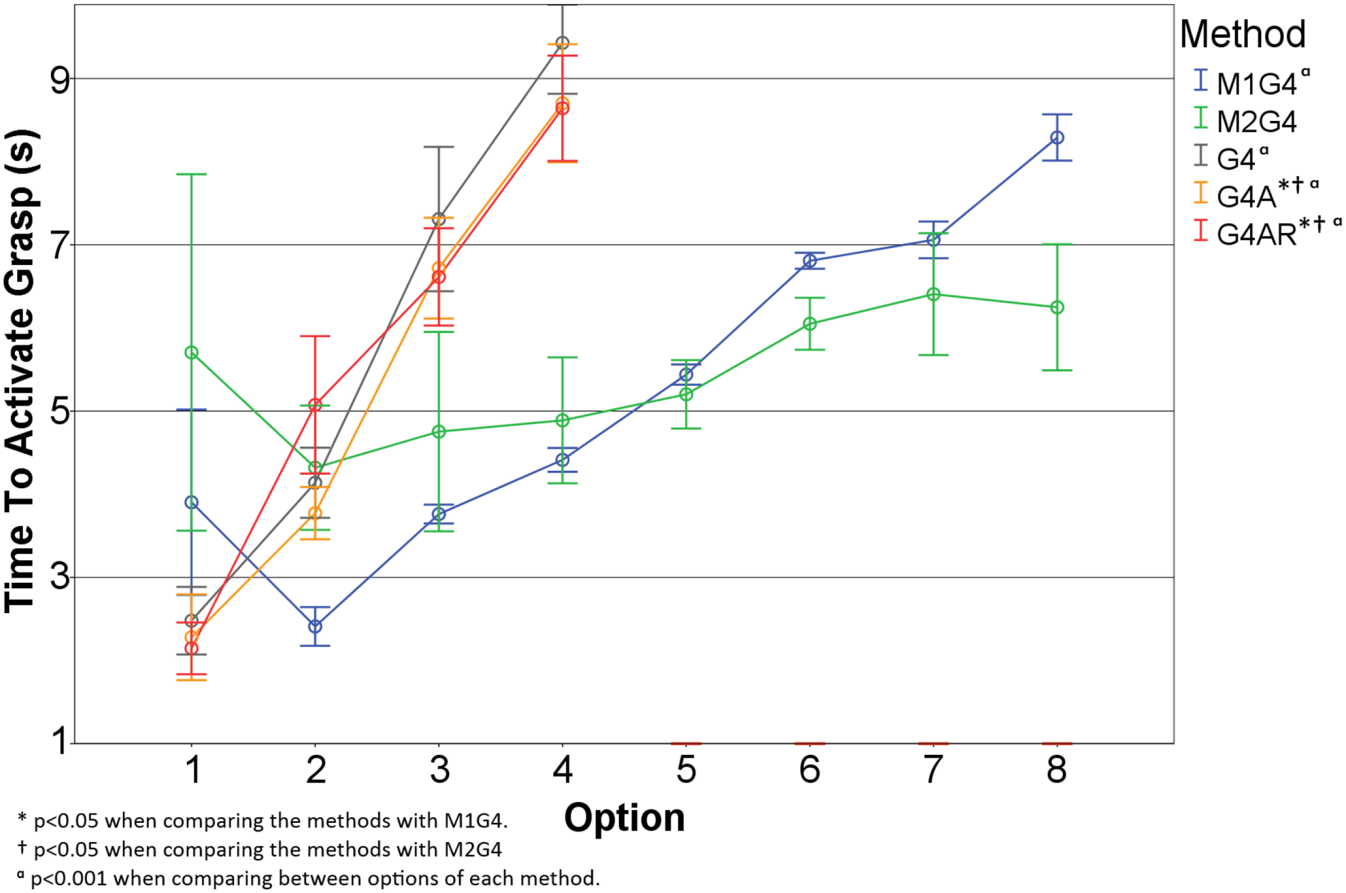
Time to Activate a Grasp Option for each of the methods used in this paper. The error bars indicate the standard error.

In Fig 5, the TTA for the different grasp options is shown. There was no significant difference between Mode 1 and Mode 2. The first option was the most difficult for subjects to select in both modes, since this was the option presented shortly after activating the system. Instead of reacting fast to trigger the option as soon as it was available, some subjects opted for a more conservative approach and often decided to wait for an additional cycle before activating the grasp. This is even more accentuated in Mode 2.

Although there were no statistically significant differences between M1G4 and M2G4, the TTA plots exhibit the trend that seems to be in accordance with our initial hypothesis that Mode 2 should allow for faster option selection (see eqs (1) and (2)). Average TTA in Mode 2 was lower for the options 6 to 8 compared to Mode 1. However, for the first 4 options, the average TTA in Mode 2 was longer than expected and also higher than in Mode 1. From the high TTA variability in these cases, it seems that the subjects were more hesitant to activate a grasp in this mode, and thus waited more often for an additional cycle before actually activating the grasp.

### Comparison between the Menu Interface and the Myocontrol

The TTA plots for Menu and Myoelectric interfaces exhibit different trends. Statistically significant differences were found for option 2, 3 and 4 between the methods, as shown in Table 4. Importantly, the selection using EMI in M1G4 was consistently faster for these options compared to all three myoelectric control setups. For M2G4, there is a similar trend, but the difference was statistically significant only for the last option. Only when activating the first option, the myoelectric interface resulted in shorter mean time, but the difference was not statistically significant.

**Table 4:** Wilcoxon signed-rank test results of the tta for each option between the two methods.

Method	M1G4	M2G4
G4	Option 2 (p<0.05, r = 0.89) Option 3 (p<0.05, r = 0.89) Option 4 (p<0.05, r = 0.89)	Option 4 (p<0.05, r = 0.89)
G4A	Option 2 (p<0.05, r = 0.89) Option 3 (p<0.05, r = 0.89) Option 4 (p<0.05, r = 0.89)	Option 4 (p<0.05, r = 0.89)
G4AR	Option 2 (p<0.05, r = 0.89) Option 3 (p<0.05, r = 0.81) Option 4 (p<0.05, r = 0.89)	Option 4 (p<0.05, r = 0.89)

A significant difference (Friedman’s ANOVA p<0.05) was also found when comparing TTA between different options within the same condition. The post hoc Bonferroni test results are shown in Table 5. For the Myoelectric interface a statistical difference was found between non-neighbouring options (1 vs. 3 and 2 vs. 4). For the EMI in Mode 1, the first significant difference between the options appeared much later (1 vs. 8, 2 vs. 6, and 3 vs. 8). This indicates that the EMI was significantly more time efficient for selecting grasps compared to myocontrol. Also, no statistical difference was found between the options in M2G4, which implies that for the systems with many options Mode 2 could be more efficient than Mode 1.

**Table 5:** Friedman’s ANOVA Post-Hoc results pair-wise comparison between options for each method.

Method	Option 1	Option 2	Option 3
Option 3	G4 (p<0.05, r = 0.67) G4A (p<0.05, r = 0.67)	—	—
Option 4	G4 (p<0.01, r = 0.89) G4A (p<0.01, r = 0.89) G4AR (p<0.001, r = 0.99)	G4 (p<0.05, r = 0.67) G4A (p<0.05, r = 0.67)	—
Option 6	—	M1G4 (p<0.05, r = 0.41)	—
Option 7	—	M1G4 (p<0.01, r = 0.41)	—
Option 8	M1G4 (p<0.05, r = 0.43)	M1G4 (p<0.01, r = 0.54)	M1G4 (p<0.05, r = 0.43)

When using the Myoelectric interface the average time (*T*_*cc*_) needed to perform a successful CoCo was approximately 2 s, more precisely, 2.34±0.66 s in G4, 2.15±0.7 s in G4A, and 2.26±0.67 s in G4AR. Importantly, this was longer then the predefined waiting time between the menu options in EMI (*T*_*D*_ = 1 s). In order for the system to detect successive CoCos, they had to be separated by brief periods of relaxation, and sometimes the subject had to perform several attempts before the system acknowledged the CoCo. The latter could be decreased through subject training. Given the values of *T*_*cc*_ and *T*_*d*_, according to the eqs (1) and (3) the selection of options using EMI in mode 1 should have been approximately twice faster for all the options. This is indeed valid for the options 2, 3 and 4 (Fig 5). However, for option 1, the myoelectric selection was in fact faster than EMI. As explained before, the time to select option 1 with EMI was prolonged since the subjects often opted to wait for one full cycle before triggering.

**Fig 6:**
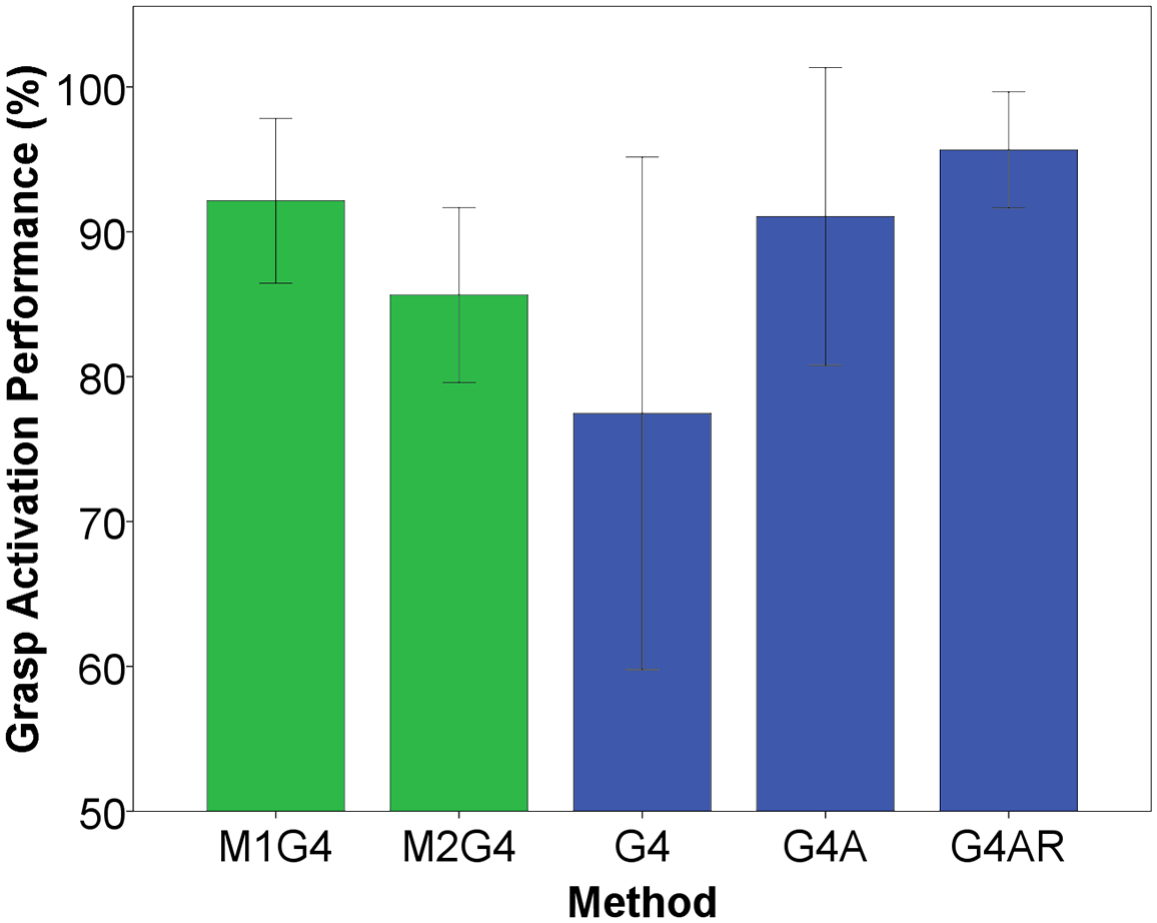
Grasp selection performance between the Menu interface modes (M1G4, M2G4) and the Myoelectric interface modes for 4 grasps (G4, G4A, G4AR). The error bars indicate the standard error.

Fig 6 compares the GSP in different conditions. When no feedback and no resetting was used (G4) in the Myoelectric interface, the GSP was lower compared to all the other conditions, although no statistical difference was found mainly due to a large variability in G4. When comparing the EMI modes (M1G4 and M2G4) to the Myoelectric modes (G4A and G4AR) the results were similar and no significant differences were found. This is an important outcome showing that the subjects could select the desired grasp with the novel method with similar success rate as when using the classical approach. The results in Table 6 show an overall comparison (pooled data) between the methods for the GA, GP and RA outcome measures. The EMI for grasping and releasing was comparable in performance to the Myoelectric interface, with the exception of the release action where subjects required significantly more attempts (p<0.05, r = 0.33) to successfully release an object using the EMI. This is not surprising since synchronizing the elbow extension with the electrocutaneous pattern pacing the release action is more difficult than just activating the extensor muscles to open the hand.

**Table 6:** Mean values (and standard deviation) of the grasp and release performance obtained during experiments with the Menu Interface and the Myocontrol Interface.

	Menu Interface	Myocontrol Interface
Grasp Attempts	1.1(0.1)	1.2(0.1)
Grasp Performance (%)	89.3(10.2)	91.31(9.9)
Release Attempts	1.2(0.2)	1.07(0.1)*

*p < 0.05

**Fig 7:**
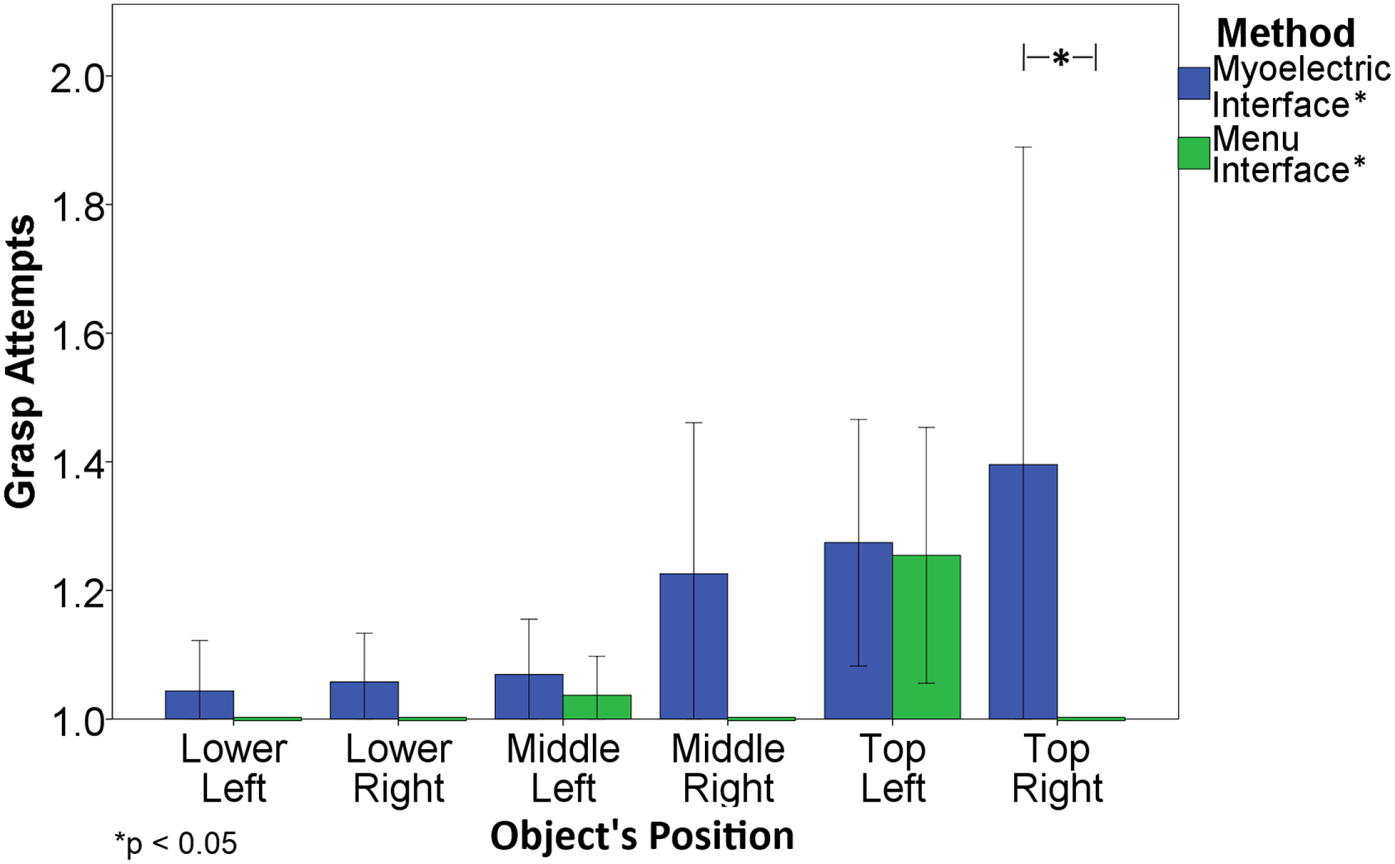
GA performance results for the different object’s position. 1 grasp attempt was the minimum possible value for this measurement. The error bars indicate the standard error.

Fig 7 shows the results for GA when target objects were placed in different position. The subjects had more difficulties to lock the grasp with the Myoelectric interface when the object was in the top position (Friedman’s ANOVA results; p < 0.05). A post-hoc test showed a significant difference between the Lower Left and the Top Right positions (p<0.05, r = 0.44), the Lower Left and the Top Left positions (p<0.05, r = 0.41), the Lower Right and the Top Right positions (p<0.05, r = 0.38), the Middle Left and the Top Left (p<0.05, r = 0.37), and the Middle Left and the Top Right (p<0.05, r = 0.39). When the subjects were using the EMI, the performance in all positions was similar with the exception of the Top Left position, for which the GA was significantly different compared to the other positions (Friedman’s ANOVA results; p < 0.05). A post-hoc test revealed a significant difference between the Top Left and the Lower Left, Lower Right, Middle Right, and Top Right positions (p<0.05, r = 0.40). There is a trend indicating a potential advantage of EMI, i.e., the mean values for EMI are consistently lower compared to myocontrol for all the positions but due to the high variability the results were statistically significantly different only for the Top Right position (Related-samples Wilcoxon signed rank test, p<0.05, r = 0.59).

## Discussion

Here we presented the first prototype to test a novel concept for a simple and transparent general-purpose HMI. The central novelty is a tactile menu system presenting the available options to the user and then waiting for an acknowledgement (selection), instead of relying on the user to generate the control signals. The proposed HMI is an easy method to increase the number of system functions without concomitantly increasing the number of command signals the user needs to generate. In this study, specifically, we were able to trigger up to 8 functions (4 grasp types and 2 grasp sizes) with a single command (reaching movement). We also demonstrated how the approach could be used to implement a two-level menu of functions (e.g. Mode 2 of the menu interface), which can be easily extended to multi-level structures of higher complexity without substantially increasing the waiting times. Certainly, there will be limits to this extension reflecting the inherent characteristics of the tactile interface as well as cognitive factors, which are discussed below in more detail.

The proposed concept simplifies the communication interface between the user and the system as well as the signal processing; instead of generating and decoding many signal patterns, only a single acknowledgement command needs to be provided by the user and recognized by the system. In general, this method is a simple approach to increase the information capacity of the existing HMIs, especially the ones that are characterized with a low bandwidth (e.g., brain computer interfacing). If the user can produce N discriminable signals (N≥1) and the tactile interface presents M states (M≥2), the total number of selectable functions becomes N x M for the 1-level menu, and can increase even further using multi-level menu structures.

The proposed concept has general applicability and can be integrated into existing control schemes. For example, in prosthetics, the EMI could be used in combination with the classic myoelectric control. In this context, myoelectric activity can be employed as trigger signals for option selection. An extensor activation could trigger the hand to assume a certain grasp type and size corresponding to the currently active option. Also, the other functions such as closing and opening could be the responsibility of the myocontrol (as in classical approach). In order for the EMI to be integrated with myocontrol, the problem of interference between the recording and stimulation has to be addressed. There are several methods that can be used for this purpose, such as, hardware and software blanking [37] but also time division multiplexing [38]. For example, the latter could be easily implemented for option selection: stimulation is presented briefly, indicating the active option, followed by a recording period (without stimulation) to detect the myoelectric trigger. This integration was outside the scope of the current study, but will be investigated as a part of the future developments.

Alternatively, the EMI does not have to be used necessarily for control, as in this study. Instead, the tactile menu could be activated by the user and employed to setup system parameters, configuring the device on the fly (e.g., change the myoelectric gains while walking). In a completely different domain, the EMI could be applied to the control of a wheelchair. The options might represent the direction to move (left, right, forward and backward) and, with a 2-level menu, even the speed of movement could be selectable. The control based on the tactile menu can be combined with the computer vision algorithms providing the obstacle detection and avoidance. In this context and also in many other applications, the proposed HMI could be of particular interest for the users with a high-level of disability. It would allow them to trigger many functions or even more complex programs (e.g., navigate the wheelchair to the end of the corridor) by generating a single control signal (e.g., a simple button implemented as a mouthpiece).

The menu based HMI presented in this study exhibits has some similarity to the event related P300 paradigm in brain computer interfacing (BCI), but there are also important differences both conceptually and practically. Whereas in these BCI systems the subject selects an option indirectly by focusing attention to a specific stimuli to evoke/modulate an involuntary neural response, in the novel HMI the stimuli delivered by the system represents only a cue prompting the subject to perform a voluntary command. In the P300 system, the neural response needs to be detected from the noisy brain signals by using specialized BCI hardware and decoding algorithms. This complicates the practical implementation of the HMI and can make it more susceptible to environmental and subjective factors, which are known drawbacks of all BCI systems [13]. Furthermore, in order to trigger a P300 response, the stimulation points have to be arranged in a specific spatial configuration and activated according to a specific protocol, while the user needs to remain focused on the desired stimulus [13].

In the novel concept, there are no such constraints, since the tactile stimulus is not used for detection. The stimuli do not need to have special characteristics in order to evoke responses that can be detected and discriminated by the system. The only requirement is that the stimuli are perceptually discriminable by the user (not by an algorithm from EEG responses). In this, the system can exploit human cognitive capabilities, such as high sensory discrimination capacity and the ability to further improve it through learning. [39]. For example, multiple commands could be triggered using a simple configuration where a single electrode is activated at different frequencies and/or intensities. Similar flexibility holds for the voluntary trigger signal, which can be selected from multiple modalities (e.g., motion, EMG) so that it is easily detectable and also related to a specific application (e.g. starting the reaching movement). Finally, the novel HMI concept as implemented in the present study is not a BCI system, but the concept could be exploited in this field as well. For example, the voluntary trigger signal for selecting options could be provided by the brain activity (e.g., motor imagery [12]).”

The fact that the menu was implemented using tactile stimulation has obvious advantages, such as, the silent operation and the fact that vision and auditory senses can be allocated to other tasks (e.g., contrary to visual P300). However, there are also certain limitations reflecting the inherent characteristics of the tactile channel, especially considering the number of options that can be implemented within the menu system. For example, only five to six frequency or intensity levels per electrode can be classified reliably by the subjects [40], [34], [41]. Multiple electrodes [42] (spatial coding) can be used, as done in the current study, and even the number of pulses comprising the stimulation burst can be a feature discriminating the stimuli, as demonstrated recently in [43]. Furthermore, the perceptual phenomena, such as habituation and tactile masking [33], has to be considered when designing the electrotactile menu system, since they can temporarily decrease the tactile sensitivity. Importantly, these effects can be minimized by properly configuring the system, for example, using lower frequencies [33], introducing pauses between the successive stimuli [44] and ensuring enough spatial separation between the electrodes [42]. However, these countermeasures can reflect on the system compactness and time efficiency by limiting the interface size and the pace of option presentation. A cognitive factor might be an additional constraint when increasing the complexity of the menu (number of options/levels), as the user has to remember the mapping between the tactile patterns and the respective commands they represent. Again, this task can be facilitated by implementing intuitive coding schemes (e.g., larger aperture represented using higher intensity) and by training the subject. For example, the subject could be provided by a visual representation of the tactile menu on the computer screen simultaneously while receiving the stimulation. Once the subject acquires the mapping between the tactile stimuli and the corresponding options, the visual feedback would be removed. Overall, the aim of this study was to present the concept and demonstrate the feasibility, while additional research is needed to define the limits of the approach.

The novel concept was used in the current study to develop a control system accommodating all the phases of a prosthetic hand operation (i.e., grasp selection, closing, closed loop grasp force control, manipulation, and opening) using simple processing and without the need of any myoelectric control. The overall result was a simple HMI that was easily comprehended by the subjects (short training) and that could accommodate many commands while still demonstrating a robust performance comparable to that of a conventional EMG driven approach.

The developed HMI relied only on one accelerometer and gyroscope sensor to detect different stages of the movement. The sensor data processing was implemented by using a simple set of rules and predefined thresholds that detected specific arm motions, which are normally encountered in reaching and grasping movements, making the system natural and intuitive to use. The experiments demonstrated that the data processing method was surprisingly robust since the same rules and thresholds were used for all of the subjects allowing them to reach objects placed at different positions.

K. Dermitzakis et al. in [45] used inertial sensors to control a hand prosthesis. They controlled different grasp types using a gyroscope and dynamic time wrapping to classify hand gestures. Although this approach is interesting, to select different options the user had to perform non-natural movements in order for the inertial sensors to produce discriminable signal patterns. In general, compared to the other non-conventional approaches [46–51] for prosthesis control, the advantage of our approach is that the system can be used more naturally and with less computation.

As shown by the experimental results, using EMI the subjects were able to achieve a high performance when selecting among eight menu options. The subjects performed better using Mode 1 (see GSP in Table 2). It seems that the two-step selection (Mode 2) was less intuitive than the direct approach (Mode 1). Also, using Mode 1 was more time efficient than Mode 2 when the number of options was not large (e.g. less than 4 options). However, when the number of options increased, the efficiency of Mode 2 becomes similar to Mode 1. It is likely that with more training and experience subjects would improve their performance in Mode 2. Similar results were obtained with the amputee, who also performed better in Mode 1. He reported that the EMI was easy to learn and use, which was as well confirmed by the good performance in both modes. This is encouraging since it implies that the system could be well accepted by amputees. However tests with more amputees have to be done using an improved EMI. In addition, it is important to investigate further how the more complex menu structures would affect the subjects’ ability to navigate the menu and trigger the desired options, and if this will increase considerably the training time.

Furthermore, the subjects’ grasp selection performance with the novel system was similar to the one obtained with the state of the art commercial myoelectric control system for the Michelangelo hand (Fig 6). Also, the performance with the EMI remained similar when the number of available grasps increased from 4 to 8 options (Table 2). Therefore, if the capabilities of the controlled system increase, the same EMI can be used simply by incorporating new options.

Interestingly, the time needed by the subjects to activate the grasp was significantly lower for the EMI compared to the Myoelectric interface for all options except the first. Therefore, contrary to our expectations, having a fixed time for choosing the grasp aided the subject to operate the system faster. It is important to note that some of the tested subjects were able to use the system with the lower waiting times than the ones used in the main experiment, i.e., 1 s for switching between the options. Namely, in three subjects, the switching time was decreased to as low as 0.5 s during extra testing after the experiment sessions and they were still able to successfully operate the prosthesis. This implies that if implemented in true real time (embedded system vs. Matlab) and after providing longer training to the subjects, the proposed HMI could be used at a significantly higher pace leading to a substantially faster operation of the system. In theory, the lower limit for the switching time between the options would be the subject’s reaction time to electrocutaneous stimuli. However, it is important to assess systematically in future experiments the effects of training, as well as the subject’s cognitive load, and attention demands. We expect that attending to the cyclic tactile menu initially requires more attention, but after some training this process could become automatized (e.g. as when processing and reacting to the feedback cues when driving a car).

When no feedback was used during the Myoelectric interface (G4), the average performance dropped and the variability between subjects was very high. Since there was no feedback to signal that a CoCo was not recognized by the system, the subject’s performance (GSP) depended on his/her individual ability to generate discriminable CoCos and track the current grasp. This result implies that it could be difficult to realize a “silent” selection with the classical myoelectric control, especially when there are more than two options. On the other side, with the EMI, tracking the currently active grasp was trivial since the grasps were presented by the menu.

Importantly, a grasp type and size selection in EMI was done using a natural reaching and grasping movement. Just by reaching for an object, the user would trigger the system and the hand would automatically preshape and start closing. The opening, transport and closing were therefore evolving simultaneously as in the normal movement. Then, just by lifting the object using a short elbow flexion, the user would lock the grasping force and the object could then be manipulated. This resulted in a continuous, smooth and natural movement pattern, which was intuitive and therefore simple for the subjects to learn. On the other hand, during the myoelectric control the movement pattern was not as smooth and natural, since most of the subjects first moved the hand in front of the object and then activated the hand closing to grasp the object (i.e., simultaneous vs. sequential reaching and grasping). For some of the large objects, the subjects had to open the hand first before closing to grasp. Also, the performance (GA) of EMI was very good for all positions, except for objects placed in the Top Left position, whereas the Myoelectric interface was less robust since the performance was significantly decreased for both top positions.

In order to release an object, subjects had significantly more difficulties when using the EMI compared to myoelectric interface. This is not surprising since it is more difficult to synchronize the elbow extension with the electrocutaneous pattern than just activating the extensor muscles to open the hand. However, the performance was relatively good considering the short training period. Although the approach used for locking and unlocking the grasp performed well for the task of lifting and releasing an object, this method is not generally applicable. For example, it could not be used for manipulating the stationary objects (a doorknob). However, the main aim of the present study was to demonstrate the potential of the novel HMI, by illustrating that it can be exploited in different contexts, rather than to develop a general, standalone, ready-to-be-applied control system.

Overall, the presented HMI was characterized with good performance, robustness, naturalness, and low user effort. Our final goal was not to replace the existing myoelectric control or other conventional HMI systems, but rather to find novel, natural and simple methods that can be used to complement and improve them. That is to say that only a part of the presented functionality (e.g., option selection), most useful for the specific application (e.g., prosthesis control), could be integrated into the other frameworks.

Finally, as discussed previously, the proposed HMI is flexible and general. The menu-based HMI could be used for the control or parameter setup in hand but also full arm prosthesis as well as in many other applications (e.g. rehabilitation robotics, functional electrical stimulation, human-computer interaction, etc.), and the selection procedure could be as well implemented in very different ways, i.e., the acknowledgement signal could be provided by electromyography, electroencephalography [18,50,51], etc. Similarly, any available stimulation technology could be used to implement the menu/feedback interface (vibrotactile stimulation [42,52–54], auditory feedback [19,55]). There is also the possibility of using different coding schemes [33,34] to represent the options to the user, thereby regulating the trade-off between the number of electrodes and ease of discrimination (e.g., parameter coding vs. spatial coding).

## Conclusion

We have demonstrated a proof of concept of a novel HMI system (EMI) that can increase the effective number of functions a user can control without concomitantly increasing the number of command signals he/she needs to generate. The HMI have a general applicability and the current study demonstrated how it could be used for controlling a multi-grasp prosthetic hand in a closed-loop manner. The system was tested with 13 healthy subjects and one amputee. The results have shown that a comprehensive control of the hand prosthesis could be successfully achieved by a completely different and new approach from the classically adopted methods. For example, after a very short training, the subjects were able to successfully operate the prosthesis, achieving very good performance. Also, it was shown that the performance and robustness of this novel system are comparable or better, in some aspects, than the classic myoelectric interfaces. The novel HMI relied on a simple processing, i.e., a rule-based state machine activated by one IMU and electrotactile stimulation. When using the EMI, the activation time of grasp selection/activation was shorter than when using the myoelectric interface; and the EMI was more robust when grasping objects in different positions. Nevertheless, it is important to emphasize that the aim of the study was not present a substitute for the commercial myocontrol. Rather, the favourable results should be regarded as a strong indication that this approach is feasible and might indeed enhance existing control schemes and HMIs if integrated into those frameworks. We envision that the potential applications are numerous and this will be explored in the future studies.

## Appendix

### 1) System activation phase (ACTIVATION)

$$sup=True,\;if\;(g_{x}<th_{gx1})\;\&\;[\;(S_{curr}=OFF)\;|\;(S_{curr}=GR\_DISP)]$$ (5)

$$S_{new}=\{\begin{array}{c} GR\_DISP,\;if\;(g_{x}>th_{gx2})\;\&\;(sup=True)\;\&\;(S_{curr}=OFF)\\ OFF,\;if\;(g_{x}>th_{gx2})\;\&\;(sup=True)\;\&\;(S_{curr}=GR\_DISP) \end{array}$$ (6)

*sup* stands for “supination”, *GR_DISP* is the state GRASP_DISPLAY, *S*_*curr*_ is the current state, *S*_*new*_ is the new state, *g*_*x*_ is the value of the gyro in the x axis, *th*_*gx1*_ and *th*_*gx2*_ are the thresholds used to decide when a supination and a pronation rotation happened.

### 2) Grasp type and size selection (GRASP_SELECTION)

Mode 1 (single step selection):

$$S_{new}=\{\begin{array}{c} RE,\;if\;(tilt_{x}>th_{ax1})\;\&\;(\frac{g_{y}+g_{z}}{2}<th_{gyz1})\;\&\;(S_{curr}=GR\_DISP)\\ GR\_DISP,\;if\;(tilt_{x}>th_{ax1})\;\&\;(\frac{g_{y}+g_{z}}{2}<th_{gyz1})\;\&\;(S_{curr}=RE) \end{array}$$ (7)

*RE* is the state REACHING, *GR_DISP* is the state GRASP_DISPLAY, *S*_*curr*_ is the current state, *S*_*new*_ is the new state, *g*_*y*_ and *g*_*z*_ are the value of the gyro in the y and z axis respectively. *th*_*ax1*_ is the threshold set to check when the arm can be considered tilted (x axis of the accelerometer). Also, *th*_*gyz1*_ is the threshold that checks if the movement can be considered to be a reaching motion.

Mode 2 (two step selection):

$$S_{new}=\{\begin{array}{c} S_{DISP},\;if\;(tilt_{x}>th_{ax2})\;\&\;(\frac{g_{y}+g_{z}}{2}<th_{gyz2})\;\&\;(S_{curr}=GR\_DISP)\\ GR\_DISP,\;if\;(tilt_{x}>th_{ax2})\;\&\;(\frac{g_{y}+g_{z}}{2}<th_{gyz2})\;\&\;(S_{curr}=S\_DISP) \end{array}$$ (8)

*S_DISP* is the state SIZE_DISPLAY, *GR_DISP* is the state GRASP_DISPLAY, *S*_*curr*_ is the current state, *S*_*new*_ is the new state, *g*_*y*_ and *g*_*z*_ are the value of the gyro in the y and z axis respectively. *th*_*ax2*_ is the threshold set to check when the arm can be considered tilted (x axis of the accelerometer). Also, *th*_*gyz1*_ is the threshold that checks if the movement can be considered to be a reaching motion.

$$S_{new}=\{\begin{array}{c} RE,\;if\;\left[\sqrt{\frac{1}{N-1}\sum_{k=1}^{N}{\left(acc-{\bar{acc}}_{x}\right)}^{2}}\right]\;\&\;(s_{curr}=GR\_DISP)\\ GR\_DISP,\;if\;(tilt_{x}>th_{ax2})\;\&\;(\frac{g_{y}+g_{z}}{2}<th_{gyz2})\;\&\;(S_{curr}=RE) \end{array}$$ (9)

*RE* is the state REACHING, *GR_DISP* is the state GRASP_DISPLAY, *S*_*curr*_ is the current state, *S*_*new*_ is the new state, *g*_*y*_ *and g*_*z*_ are the value of the gyro in the y and z axis respectively. *th*_*ax2*_ is the threshold set to check when the arm can be considered tilted (x axis of the accelerometer). Also, *th*_*gyz2*_ is the threshold that checks if the movement can be considered to be a reaching motion. *th*_*sd1*_ is the threshold used to detect a variation in the acceleration (standard deviation). Also *N* is the number of points used to calculate the standard deviation from the accelerometer x-axis (*acc*_*x*_).

### 3) Reaching and grasping (REACH_AND_GRASP)

$$S_{new}=GR,\;if\;(g_{y}<th_{gy2})\;\&\;(g_{z}<th_{gz2})\;\&\;(S_{curr}=RE)$$ (10)

*GR* is the state GRASP, *S*_*curr*_ is the current state, *S*_*new*_ is the new state, *g*_*y*_ *and g*_*z*_ are the value of the gyro in the y and z axis respectively. *th*_*gy2*_ and *th*_*gz2*_ are the thresholds used to detect elbow flexion.

### 4) Release phase (RELEASE)

$$S_{new}=GRe,\;if\;(\frac{g_{y}+g_{z}}{2}>th_{gyz2})\;\&\;(S_{curr}=GR)$$ (11)

*GRe* is the state RELEASE, *S*_*curr*_ is the current state, *S*_*new*_ is the new state, *g*_*y*_ and *g*_*z*_ are the value of the gyro in the y and z axis respectively. *th*_*gyz2*_ was used to detect an elbow flexion.

## References

[1] Heo P, Gu GM, Lee S, Rhee K, Kim J (2012) Current hand exoskeleton technologies for rehabilitation and assistive engineering. Int J Precis Eng Manuf 13: 807–824. 10.1007/s12541-012-0107-2

[2] Lo HS, Xie SQ (2012) Exoskeleton robots for upper-limb rehabilitation: state of the art and future prospects. Med Eng Phys 34: 261–268. Available: http://www.sciencedirect.com/science/article/pii/S1350453311002694. Accessed: 2014 Mar 26. 10.1016/j.medengphy.2011.10.004 22051085

[3] Pedrocchi A, Ferrante S, Ambrosini E, Gandolla M, Casellato C, Schauer T, et al (2013) MUNDUS project: Multimodal Neuroprosthesis for daily Upper limb Support. J Neuroeng Rehabil 10: 66 Available: http://www.pubmedcentral.nih.gov/articlerender.fcgi?artid=3733825&tool=pmcentrez&rendertype=abstract. Accessed: 2014 Jan 15. 10.1186/1743-0003-10-66 23822118 PMC3733825

[4] Schill O, Wiegand R, Schmitz B, Matthies R, Eck U, Pylatiuk C, et al (2011) OrthoJacket: an active FES-hybrid orthosis for the paralysed upper extremity. Biomed Tech (Berl) 56: 35–44. 10.1515/BMT.2010.056 21210758

[5] Belter JT, Segil JL, Dollar AM, Weir RF (2013) Mechanical design and performance specifications of anthropomorphic prosthetic hands: a review. J Rehabil Res Dev 50: 599–618. 24013909 10.1682/jrrd.2011.10.0188

[6] Bebionic Hand (2014). Available: http://bebionic.com/.

[7] Touch Bionics (2014). Available: http://www.touchbionics.com/.

[8] Resnik L, Klinger SL, Etter K (2013) The DEKA Arm: Its features, functionality, and evolution during the Veterans Affairs Study to optimize the DEKA Arm. Prosthet Orthot Int: 0309364613506913 –. 10.1177/0309364613506913 24150930

[9] Jiang N, Falla D, d’Avella A, Graimann B, Farina D (2010) Myoelectric control in neurorehabilitation. Crit Rev Biomed Eng 38: 381–391. 21133839 10.1615/critrevbiomedeng.v38.i4.30

[10] Kübler A, Neumann N (2005) Brain-computer interfaces—the key for the conscious brain locked into a paralyzed body. Prog Brain Res 150: 513–525. 10.1016/S0079-6123(05)50035-9 16186045

[11] Kuiken TA, Li G, Lock BA, Lipschutz RD, Miller LA, Stubblefield K, et al (2009) Targeted muscle reinnervation for real-time myoelectric control of multifunction artificial arms. JAMA 301: 619–628. 10.1001/jama.2009.116 19211469 PMC3036162

[12] Becedas J (2012) Brain—Machine Interfaces: Basis and Advances. IEEE Trans Syst Man, Cybern Part C (Applications Rev 42: 825–836. 10.1109/TSMCC.2012.2203301

[13] Nicolas-Alonso LF, Gomez-Gil J (2012) Brain computer interfaces, a review. Sensors (Basel) 12: 1211–1279. Available: http://www.pubmedcentral.nih.gov/articlerender.fcgi?artid=3304110&tool=pmcentrez&rendertype=abstract. Accessed: 2014 Oct 17. 10.3390/s120201211 22438708 PMC3304110

[14] Brouwer AM, van Erp JBF (2010) A tactile P300 brain-computer interface. Front Neurosci 4: 1–11. 10.3389/fnins.2010.00019 20582261 PMC2871714

[15] Iturrate I, Antelis JM, Kübler A, Minguez J (2009) A noninvasive brain-actuated wheelchair based on a P300 neurophysiological protocol and automated navigation. IEEE Trans Robot 25: 614–627. 10.1109/TRO.2009.2020347

[16] Ganin IP, Shishkin SL, Kaplan AY (2013) A P300-based brain-computer interface with stimuli on moving objects: four-session single-trial and triple-trial tests with a game-like task design. PLoS One 8: e77755 Available: http://dx.plos.org/10.1371/journal.pone.0077755. 10.1371/journal.pone.0077755 24302977 PMC3840230

[17] Markovic M, Dosen S, Cipriani C, Popovic D, Dario Farina (2014) Stereovision and augmented reality for closed loop control of grasping in hand prostheses. J Neural Eng. 10.1088/1741-2560/11/4/046001 24891493

[18] Ramos-Murguialday A, Schürholz M, Caggiano V, Wildgruber M, Caria A, Hammer E, et al (2012) Proprioceptive Feedback and Brain Computer Interface (BCI) Based Neuroprostheses. PLoS One 7: e47048 Available: http://www.pubmedcentral.nih.gov/articlerender.fcgi?artid=3465309&tool=pmcentrez&rendertype=abstract. Accessed: 2012 Nov 12. 10.1371/journal.pone.0047048 23071707 PMC3465309

[19] Gonzalez J, Soma H, Sekine M, Yu W (2011) Auditory Display as a Prosthetic Hand Biofeedback. J Med Imaging Heal Informatics 1: 325–333. Available: http://openurl.ingenta.com/content/xref?genre=article&issn=2156-7018&volume=1&issue=4&spage=325. Accessed: 2012 Dec 17.

[20] Hahne JM, Graimann B, Müller K-R (2012) Spatial filtering for robust myoelectric control. IEEE Trans Biomed Eng 59: 1436–1443. Available: http://www.ncbi.nlm.nih.gov/pubmed/22374342. Accessed: 2013 Aug 11. 10.1109/TBME.2012.2188799 22374342

[21] Simon AM, Hargrove LJ, Lock BA, Kuiken TA (2011) A decision-based velocity ramp for minimizing the effect of misclassifications during real-time pattern recognition control. IEEE Trans Biomed Eng 58: 2360–2368. Available: http://ieeexplore.ieee.org/articleDetails.jsp?arnumber=5768069. Accessed: 2013 Aug 14. 10.1109/TBME.2011.2155063 PMC4269322 21592916

[22] Geng Y, Zhou P, Li G (2012) Toward attenuating the impact of arm positions on electromyography pattern-recognition based motion classification in transradial amputees. J Neuroeng Rehabil 9: 74 Available: http://www.jneuroengrehab.com/content/9/1/74. Accessed: 2013 Aug 7. 10.1186/1743-0003-9-74 23036049 PMC3551659

[23] Scheme E, Englehart K (2011) Electromyogram pattern recognition for control of powered upper-limb prostheses: state of the art and challenges for clinical use. J Rehabil Res Dev 48: 643–659. Available: http://www.ncbi.nlm.nih.gov/pubmed/21938652. Accessed: 2013 Aug 14. 21938652 10.1682/jrrd.2010.09.0177

[24] Ortiz-Catalan M, Brånemark R, Håkansson B, Delbeke J (2012) On the viability of implantable electrodes for the natural control of artificial limbs: review and discussion. Biomed Eng Online 11: 33 Available: http://www.pubmedcentral.nih.gov/articlerender.fcgi?artid=3438028&tool=pmcentrez&rendertype=abstract. Accessed: 2014 Oct 6. 10.1186/1475-925X-11-33 22715940 PMC3438028

[25] Lauren H. Smith LJH (2013) Comparison of surface and intramuscular EMG pattern recognition for simultaneous wrist/hand motion classification. Conference proceedings: Annual International Conference of the IEEE Engineering in Medicine and Biology Society. IEEE Engineering in Medicine and Biology Society. Conference. pp. 4223–4226. 10.1109/EMBC.2013.6610477 PMC3914139 24110664

[26] Raspopovic S, Capogrosso M, Petrini FM, Bonizzato M, Rigosa J, Di Pino G, et al (2014) Restoring natural sensory feedback in real-time bidirectional hand prostheses. Sci Transl Med 6: 222ra19 Available: http://stm.sciencemag.org/content/6/222/222ra19.abstract. Accessed: 2014 Jul 10. 10.1126/scitranslmed.3006820 24500407

[27] Yatsenko D, McDonnall D, Guillory KS (2007) Simultaneous, proportional, multi-axis prosthesis control using multichannel surface EMG. Conference proceedings: Annual International Conference of the IEEE Engineering in Medicine and Biology Society. IEEE Engineering in Medicine and Biology Society. Conference. Vol. 2007. pp. 6134–6137. Available: http://www.ncbi.nlm.nih.gov/pubmed/18003415. Accessed: 2013 Aug 14. 10.1109/IEMBS.2007.4353749 18003415

[28] Kamavuako EN, Rosenvang JC, Horup R, Jensen W, Farina D, Englehart KB (2013) Surface Versus Untargeted Intramuscular EMG Based Classification of Simultaneous and Dynamically Changing Movements. IEEE Trans Neural Syst Rehabil Eng PP: 1 Available: http://ieeexplore.ieee.org/articleDetails.jsp?arnumber=6476027. Accessed: 2013 Aug 14. 10.1109/TNSRE.2012.2211036 23481867

[29] Jiang N, Englehart KB, Parker P (2009) Extracting simultaneous and proportional neural control information for multiple-DOF prostheses from the surface electromyographic signal. IEEE Trans Biomed Eng 56: 1070–1080. Available: http://www.ncbi.nlm.nih.gov/pubmed/19272889. 10.1109/TBME.2008.2007967 19272889

[30] Cipriani C, Zaccone F, Micera S, Carrozza MC (2008) On the Shared Control of an EMG-Controlled Prosthetic Hand: Analysis of User—Prosthesis Interaction. IEEE Trans Robot 24: 170–184. 10.1109/TRO.2007.910708

[31] Szeto a Y (1985) Relationship between pulse rate and pulse width for a constant-intensity level of electrocutaneous stimulation. Ann Biomed Eng 13: 373–383. Available: http://www.ncbi.nlm.nih.gov/pubmed/4073624. 4073624 10.1007/BF02407767

[32] Kaczmarek KA, Webster JG, Radwin RG (1992) Maximal dynamic range electrotactile stimulation waveforms. IEEE Trans Biomed Eng 39: 701–715. Available: http://www.ncbi.nlm.nih.gov/pubmed/1516937. 1516937 10.1109/10.142645

[33] Szeto A, Saunders F (1982) Electrocutaneous stimulation for sensory communication in rehabilitation engineering. IEEE Trans Biomed Eng 29: 300–308. Available: http://www.ncbi.nlm.nih.gov/pubmed/7068167. 7068167

[34] Kaczmarek KA, Webster JG, Bach-y-Rita P, Tompkins WJ (1991) Electrotactile and vibrotactile displays for sensory substitution systems. IEEE Trans Biomed Eng 38: 1–16. Available: http://www.ncbi.nlm.nih.gov/pubmed/22939849. 2026426 10.1109/10.68204

[35] Kingdom FAA, Prins N (2009) Psychophysics: A Practical Introduction. Academic Press 304 p. Available: http://www.amazon.com/Psychophysics-Introduction-Frederick-A-A-Kingdom/dp/0123736560. Accessed: 2013 Aug 14.

[36] Cutkosky MR (1989) On grasp choice, grasp models, and the design of hands for manufacturing tasks. IEEE Trans Robot Autom 5: 269–279. Available: http://ieeexplore.ieee.org/articleDetails.jsp?arnumber=34763. Accessed: 2015 Feb 10.

[37] Hartmann C, Dosen S, Amsuess S, Farina D (2014) Closed-Loop Control of Myoelectric Prostheses with Electrotactile Feedback: Influence of Blanking for Removal of the Stimulation Artifact. IEEE Trans Neural Syst Rehabil Eng in press. 10.1109/TNSRE.2014.2357175 25222951

[38] Dosen S, Schaeffer M-C, Farina D (2014) Time-division multiplexing for myoelectric closed-loop control using electrotactile feedback. J Neuroeng Rehabil 11: 138 10.1186/1743-0003-11-138 25224266 PMC4182789

[39] Flor H, Denke C, Schaefer M, Grüsser S (2001) Effect of sensory discrimination training on cortical reorganisation and phantom limb pain. Lancet 357: 1763–1764. Available: http://www.thelancet.com/article/S014067360004890X/fulltext. Accessed: 2015 Jan 27. 11403816 10.1016/S0140-6736(00)04890-X

[40] Anani AB, Ikeda K, Körner LM (1977) Human ability to discriminate various parameters in afferent electrical nerve stimulation with particular reference to prostheses sensory feedback. Med Biol Eng Comput 15: 363–373. 197328 10.1007/BF02457988

[41] D’Alonzo M, Dosen S, Cipriani C, Farina D (2013) HyVE: Hybrid Vibro-Electrotactile Stimulation for Sensory Feedback and Substitution in Rehabilitation. IEEE Trans Neural Syst Rehabil Eng 22: 290–301. Available: http://www.ncbi.nlm.nih.gov/pubmed/23782817. Accessed: 2014 Mar 14. 10.1109/TNSRE.2013.2266482 23782817

[42] D’Alonzo M, Dosen S, Cipriani C, Farina D (2013) HyVE—Hybrid Vibro-Electrotactile Stimulation—is an Efficient Approach to Multi-Channel Sensory Feedback. IEEE Trans Haptics PP: 1–1. Available: http://www.computer.org/csdl/trans/th/preprint/06615899-abs.html. Accessed: 2014 Mar 14. 10.1109/TOH.2013.52 24968382

[43] Geng B, Jensen W (2014) Human ability in identification of location and pulse number for electrocutaneous stimulation applied on the forearm. J Neuroeng Rehabil 11: 97 10.1186/1743-0003-11-97 24908048 PMC4060858

[44] Buma DG, Buitenweg JR, Veltink PH (2007) Intermittent stimulation delays adaptation to electrocutaneous sensory feedback. IEEE Trans Neural Syst Rehabil Eng 15: 435–441. Available: http://ieeexplore.ieee.org/xpl/articleDetails.jsp?tp=&arnumber=4303097&contentType=Journals+&+Magazines&searchField=Search_All&queryText=Intermittent+Stimulation+Delays+Adaptation+to+Electrocutaneous+Sensory+Feedback. Accessed: 2012 Dec 12. 17894276 10.1109/TNSRE.2007.903942

[45] Dermitzakis K, Arieta AH, Pfeifer R (2011) Gesture recognition in upper-limb prosthetics: a viability study using dynamic time warping and gyroscopes. Conference proceedings: Annual International Conference of the IEEE Engineering in Medicine and Biology Society. IEEE Engineering in Medicine and Biology Society. Conference. Vol. 2011. pp. 4530–4533. Available: http://ieeexplore.ieee.org/xpls/abs_all.jsp?arnumber=6091122’escapeXml='false'/>. Accessed: 2013 Jan 14. 10.1109/IEMBS.2011.6091122 22255345

[46] Carrozza MC, Persichetti A, Laschi C, Vecchi F, Lazzarini R, Tamburrelli V, et al. (2005) A Novel Wearable Interface for Robotic Hand Prostheses. 9th Int Conf Rehabil Robot 2005 ICORR 2005: 109–112. Available: http://ieeexplore.ieee.org/lpdocs/epic03/wrapper.htm?arnumber=1501063.

[47] Mainardi E, Davalli A (2007) Controlling a prosthetic arm with a throat microphone. Conf Proc Annu Int Conf IEEE Eng Med Biol Soc IEEE Eng Med Biol Soc Conf 2007: 3035–3039. Available: http://ieeexplore.ieee.org/articleDetails.jsp?arnumber=4352968. Accessed: 2013 Sep 16. 18002634 10.1109/IEMBS.2007.4352968

[48] Johansen D, Popović DB, Struijk LNSA, Sebelius F, Jensen S (2012) A Novel Hand Prosthesis Control Scheme Implementing a Tongue Control System. Int J Eng Manuf 2: 14 Available: http://www.mecs-press.org/ijem/ijem-v2-n5/v2n5-3.html. Accessed: 2013 Sep 16.

[49] Hao Y, Controzzi M, Cipriani C, Popovic DB, Carrozza MC (2013) Controlling hand-assistive devices: utilizing electrooculography as a substitute for vision. IEEE Robot Autom Mag 20: 40–52. Available: http://ieeexplore.ieee.org/lpdocs/epic03/wrapper.htm?arnumber=6476693. Accessed: 2013 Sep 16.

[50] Bradberry TJ, Gentili RJ, Contreras-Vidal JL (2010) Reconstructing three-dimensional hand movements from noninvasive electroencephalographic signals. J Neurosci 30: 3432–3437. Available: http://www.ncbi.nlm.nih.gov/pubmed/20203202. Accessed: 2013 Mar 7. 10.1523/JNEUROSCI.6107-09.2010 20203202 PMC6634107

[51] Onose G, Grozea C, Anghelescu A, Daia C, Sinescu CJ, Ciurea AV, et al (2012) On the feasibility of using motor imagery EEG-based brain-computer interface in chronic tetraplegics for assistive robotic arm control: a clinical test and long-term post-trial follow-up. Spinal Cord 50: 599–608. Available: http://www.ncbi.nlm.nih.gov/pubmed/22410845. Accessed: 2012 Oct 30. 10.1038/sc.2012.14 22410845

[52] Cipriani C, D’Alonzo M, Carrozza MC (2012) A miniature vibrotactile sensory substitution device for multifingered hand prosthetics. IEEE Trans Biomed Eng 59: 400–408. Available: http://www.ncbi.nlm.nih.gov/pubmed/22042125. 10.1109/TBME.2011.2173342 22042125

[53] Witteveen HJB, Droog EA, Rietman JS, Veltink PH (2012) Vibro- and electrotactile user feedback on hand opening for myoelectric forearm prostheses. IEEE Trans Biomed Eng 59: 2219–2226. Available: http://www.ncbi.nlm.nih.gov/pubmed/22645262. 10.1109/TBME.2012.2200678 22645262

[54] Antfolk C, Balkenius C (2010) A tactile display system for hand prostheses to discriminate pressure and individual finger localization. J Med Biol Eng 30: 355–360. Available: http://jmbe.bme.ncku.edu.tw/index.php/bme/article/viewArticle/543. Accessed: 2012 Dec 12.

[55] Lundborg G, Rosén B, Lindberg S (1999) Hearing as Substitution for Sensation : A New Principle for Artificial Sensibility. J Hand Surg Am: 219–224. Available: http://www.sciencedirect.com/science/article/pii/S0363502399114278. Accessed: 2012 Dec 12. 10194002 10.1053/jhsu.1999.0219

